# A theory-based behavior-change intervention to reduce alcohol consumption in undergraduate students: Trial protocol

**DOI:** 10.1186/s12889-015-1648-y

**Published:** 2015-03-31

**Authors:** Martin S Hagger, Ging Ging Wong, Simon R Davey

**Affiliations:** 1 grid.1032.00000000403754078Health Psychology and Behavioural Medicine Research Group, School of Psychology and Speech Pathology, Faculty of Health Sciences, Curtin University, GPO Box U1987, Perth, WA 6845 Australia; 2 grid.5846.f0000000121619644Department of Psychology, University of Hertfordshire, Hatfield, Hertfordshire United Kingdom

**Keywords:** Binge drinking, Self-control, Ego-depletion, Strength model, Mental simulations, Imagery, Motivation, Behavior change

## Abstract

**Background:**

Excessive alcohol consumption on single occasions among undergraduate students is a major health issue as research has shown this pattern of drinking to be related to maladaptive health and psychosocial outcomes. Brief, theory-based interventions targeting motivation and self-control as behavior-change techniques have been identified as effective means to reduce alcohol consumption, but few studies have examined the interactive effects of these components. The aim of the present study is to develop a brief theory-based intervention using motivational and self-control intervention techniques to reduce alcohol consumption in undergraduate students.

**Methods/Design:**

The intervention will adopt a factorial design to test the main and interactive effects of the techniques on alcohol consumption. Using mental simulations and the strength model of self-control as the theoretical bases of the intervention, the study will adopt a fully randomized 2 (mental simulation: mental simulation vs. control irrelevant visualization exercise) × 2 (self-control training: challenging Stroop task vs. easy Stroop task) between-participants design. Non-abstinent undergraduate students aged 18 years or older will be eligible to participate in the study. Participants will complete an initial survey including self-reported alcohol consumption measures, measures of motivation and self- measures. Participants will be randomly allocated to receive either a mental simulation exercise presented in print format or a control irrelevant visualization exercise. Thereafter, participants will be randomly assigned to receive a challenging online self-control training exercise or an easy training exercise that has little self-control demand over the course of the next four weeks. Four weeks later participants will complete a follow-up alcohol consumption, motivation and self-control measures.

**Discussion:**

This study will provide the first evidence for the individual and interactive effects of motivational and self-control training techniques in an intervention to reduce alcohol consumption. It will also demonstrate the importance of adopting multiple theoretical perspectives and a factorial design to identify the unique and interactive impact of behavior-change techniques on health behavior.

**Trial registration:**

The trial is registered with the Australian and New Zealand Clinical Trials Registry, ACTRN12613000573752.

**Electronic supplementary material:**

The online version of this article (doi:10.1186/s12889-015-1648-y) contains supplementary material, which is available to authorized users.

## Background

Australians have high rates of alcohol consumption especially among younger populations [1]. In particular, younger populations frequently engage in high-risk single-session alcohol drinking, frequently termed ‘binge’ drinking. A recent survey revealed that two out of five young drinkers regularly engage in binge drinking putting them at significantly increased risk of alcohol-related injury [1]. In addition, 31.7% of Australians aged 18 and19 years and 26.9% of Australians aged 20 to 29 years are more likely to engage in risky drinking patterns leading to an increased vulnerability to alcohol-related harm over their lifetime when compared with older age groups. In addition, excessive alcohol consumption has had a negative impact on Australian economic and social outcomes [2]. For example, treatment of alcohol-related illnesses and injuries is estimated to cost 14.352 billion Australian dollars per year [3]. Excessive alcohol consumption is also related to maladaptive health outcomes such as diabetes, heart disease, asthma, risk of cancer, mortality, and mental illness [1,4].

The problem of excessive alcohol consumption is particularly exacerbated among student populations. Rates of high-risk alcohol consumption patterns such as ‘binge’ drinking are often higher in University and college student samples compared with non-student samples of the same age [5-7]. Research has shown that undergraduate students who consume excessive alcohol are vulnerable to profound acute and chronic harms, such as drink driving, substance abuse, alcohol-related injuries, violence, and alcohol dependence [4,8,9]. Studies have also demonstrated that undergraduate students who drink excessively are more likely to perform poorly in their studies [10,11] and subsequently drop out from university [12].

A potential solution to reduce prevalence of excessive alcohol consumption among undergraduate students is the development and implementation of behavioral interventions based on social psychological theory and models of behavior change. Many interventions adopting these kinds of approaches have been developed and applied to reduce alcohol consumption [13-17]. Despite the relative success of these interventions, two problems exist. First, many of the interventions adopt multiple theories and, as a consequence, multiple intervention techniques. Although the resulting intervention may have demonstrated efficacy in changing the primary behavioral outcome variable, and passed ‘fit for purpose’ on that basis, the intervention itself is not revealing in terms of the precise mechanism by which the intervention exerts its effects and does not permit the isolation of the individual techniques so that their relative independent effectiveness on the behavioral outcome can be ascertained [18,19]. This means that the interventions are comparatively silent on exactly what components of the intervention ‘work’ in terms of changing behavior and ‘how’ the components exert their effects [20]. Recent research on the components of behavioral interventions has called for better intervention designs to isolate the individual components or *techniques* that are effective in bringing about a change in behavioral outcomes and to explain the mechanisms behind the effects through appropriate mediator variables [21-24]. As a consequence, interventions need to adopt factorial designs examining individual intervention components in isolation, and in synergy, to ensure that the independent and interactive effects of the intervention techniques can be isolated and to include measures of the psychological components that are proposed to mediate the effects of the intervention on behavioral outcomes [18,19].

Taking these considerations into account, the aim of the present study is to develop a theory-based intervention that includes multiple intervention techniques from two different social psychological theories of behavior change in a randomized-controlled factorial design to reduce alcohol consumption in undergraduate students. The intervention will include appropriate theory-based mediator variables of target components associated with the intervention techniques to explain the processes behind the proposed intervention effects. The intervention will make a unique contribution to knowledge by not only demonstrating significant reduction in the primary and secondary outcome variables, namely undergraduate alcohol consumption and binge drinking occasions, but demonstrate in independent and interactive effects of the intervention techniques on the outcome and the appropriate mediator variables. The intervention will be driven by two emerging theoretical approaches relevant to individuals’ self-regulation of behavior, namely, the *mental simulation* approach and the *strength* or *resource-depletion* model of self-control. In the following sections, we outline these theoretical approaches, highlight their relevance to reducing alcohol consumption, and introduce the intervention components and the psychological constructs that the components are hypothesized to change and, therefore, provide a mechanistic explanation of the effectiveness of the intervention.

### Imagery, mental simulations, and behavioral engagement

There has been a recent resurgence of interest in the efficacy of visualization, rehearsal and imagery strategies in promoting behavior change [25,26]. The effectiveness of imagery-based strategies stems from the tenets of Bandura’s [27,28] social cognitive theory. According to the theory, imagery, particularly imagining or visualizing the self engaging in a desired behavior, leads to behavior change because it provides a form of a ‘self-model’ or vicarious experience, which is central to the development of self-efficacy. Self-efficacy is, therefore, one potential mechanism by which such imagery strategies affect a change in behavior. Another mechanism by which imagery-based techniques may promote behavioral engagement is through promoting greater importance of the goal [25,26] and promoting better accessibility of cues to action and goals [29,30].

One class of imagery-based intervention techniques is *mental simulations*, which are defined as imagining and rehearsing future events. There are two kinds of mental simulations: outcome simulation and process simulation. Outcome simulation involves imagining attainment of a targeted goal while process simulation requires imagining and rehearsing the steps required to achieve the goal [31]. Mental simulations have been shown to be effective in evoking behavior change in diverse contexts such as studying for exams [31], fruit consumption [30], intention to buy a product [32], anxiety reduction [33], and alcohol consumption [34]. The effect sizes of mental simulation interventions is generally small-to-medium, with a medium effect size reported for the research on alcohol consumption of employees, the context that is most closely aligned to that of the current study [34]. The effect size is comparable with the effect sizes of intervention techniques designed to change intentions [35] and self-efficacy [36] in research on health behavior change.

A key approach to understanding intervention effectiveness is to identify the psychological variables that mediate the effects of interventions on behavioral outcomes [37,38]. Researchers have turned to social cognitive [39-46] and integrated models of motivation [18,47-56] in order to identify the key mediators of interventions. Research has suggested that imagery-based and mental simulation intervention techniques in health behavior exert their effects through changes in motivation [30,57], intentions and attitudes [33,58] and planning [31], although there are few studies that have conducted formal mediator analyses. Knauper et al. [30] revealed that the effectiveness of a mental imagery intervention was mediated by motivation and Pham and Taylor [31] demonstrated that planning was a key mediator of the effect of mental simulations on studying behavior, which is highly salient given increased recent interest in planning interventions in health contexts [59]. Interestingly, no studies have found self-efficacy to be a mediator of the effects of imagery on behavior despite hypothesizing it as a theoretically-relevant mediator, measuring the construct and including it as a mediator in analyses [30,33,57]. Research in other domains, such as injury prevention, have found effects for imagery interventions on self-efficacy and behavioral outcomes, implicating it in the process by which imagining processes and outcomes may effect behavior change [60,61]. Overall, research in the health domain has presented consistent evidence to support the effectiveness of imagery-based interventions such as mental simulations on health related behavior. On the proposed mechanisms, however, the evidence is less conclusive with motivation and self-efficacy as identified as possible mechanisms for mental simulation effects.

### The ‘Strength’ or ‘Resource Depletion’ Model of Self-control

Self-control is another construct that has been identified as an important factor associated with the regulation of health-related behavior [62-64]. Self-control is defined as the capacity to control or regulate impulses, temptations, or ‘dominant’ responses and to overcome well-learned, ingrained habitual actions for some goal-directed alternative [65-72]. Much of the research on self-control in health domains has focused on trait conceptualizations of self-control and has demonstrated that good’ self-control is associated with numerous adaptive health-related behaviors and outcomes [62,73-77].

An alternative perspective on self-control is offered by the ‘strength’ or resource depletion model which conceptualizes self-control as limited resource that permits individuals to engage in acts of self-control, but only for a finite period after which the resource becomes depleted leading to impaired self-control capacity unless an individual is able to rest and recover [64,78-80]. The state of reduced self-control capacity or ‘strength’ is known as *ego-depletion*. Research adopting the model has typically adopted an experimental procedure, known as the dual task paradigm, to test model effects [78,81]. The paradigm requires individuals to engage in two consecutive tasks, for experimental group participants both tasks require self-control while for control group participants only the second task requires self-control. To the extent that experimental participants’ performance on the second self-control task is impaired, we have sharp confirmation of the ego-depletion effect. Research has supported the ego-depletion effect across multiple studies and has shown that the depletion effect occurs for tasks in multiple domains of self-control indicating that the resource is a unitary, generalized effect rather one that is confined to particular tasks [63,70,81-83].

An important additional hypothesis derived from the strength model is the training effect. A growing number of studies have demonstrated that repeated practice on self-control tasks improves regulatory capacity. According to the strength model, engaging in tasks that demand self-control on a regular basis can improve self-control capacity by ‘building up’ additional resources that can be made available or by making the application of the resource more efficient [84]. Research has demonstrated that regular practice on self-control tasks in laboratory and field settings leads to better performance on self-control tasks in the laboratory [84-86] as well as health-related behaviors requiring self-control [87-90] including alcohol consumption [91-93]. The strength of self-control training effects has been shown to be of medium effect size which is comparable to other interventions such as those targeting intention [35] and self-efficacy [36]. For example, a meta-analysis of self-control training on self-control task performance found a medium-sized effect [81] and a recent meta-analysis of response-inhibition training, using similar tasks to those used in the current study, on health behavior found a small-to-medium sized effect [94].

Training studies have demonstrated that the practice of self-control promotes behavior change in a number of contexts, providing indication of the generalized, unitary nature of self-control resources [85,87,93]. However, a key unresolved issue is the mechanism that drives the direction and allocation of self-control resources to increase behavioral enactment. It is unlikely that individuals will commit self-control resources toward engaging in behaviors for which they have no motivation. The resources would more likely be allocated elsewhere, such as toward enacting behaviors that they are motivated to perform. This gives rise to the possibility is that behavior change will be more effective if participants can direct their self-control efforts towards a particular target behavior for which they are highly motivated [95,96]. Interventions might, therefore, be more effective if means to increase motivation toward behaviors could be delivered alongside the practice on self-control tasks in a factorial design giving participants the opportunity and drive to direct their self-control resources toward that specific behavior. Instilling increased motivation may, therefore, be effective in focusing individuals on directing their self-control resources towards specific target behavior.

### The current research

The purpose of the current research is to develop a brief theory-based intervention adopting imagery-based motivational and self-control training behavior-change components and a randomized controlled factorial-design that will lead to a reduction in alcohol consumption among undergraduate students, an at-risk population, over a four-week period. We expect that the influence of motivational and self-control components on promoting alcohol reduction will interact, such that individuals who are both motivated and provided with training to enhance their self-regulatory capacity will exhibit the greatest reduction in their alcohol consumption. Why would training self-control improve an individual’s capacity to reduce their future alcohol consumption? Our position is that training individuals to inhibit well-learned, ingrained responses with little cognitive control or conscious thought (i.e., so called ‘automatic’ or ‘habitual’ behaviors) will have abroad impact on behaviors that are dependent on such automatic processes [69,72]. This proposition is consistent with recent trends in theory on behavioral enactment which indicate that actions are controlled by two systems: reflective and impulsive [97]. The reflective system is a deliberative pathway to action in which individuals decide on a course of action as a result of conscious consideration of the costs, benefits, consequences and outcomes of the action. Such a system is controlled, slow, reasoned, and conscious, often termed a ‘cool’ system by some theorists [98]. This is contrasted with the impulsive system which is a more spontaneous, automatic pathway to action in which individuals act in response to well-learned cues or heuristics that require little conscious involvement or deliberation. The impulsive system is fast, non-conscious, and automated and often referred to as a ‘hot’ system [98].

As actions controlled by the impulsive system often occur outside the individual’s awareness and in responses to well-learned cue-response pairings, it is difficult to override and change such actions i.e. to break the cue-response link. It often takes considerable self-control or capacity to inhibit responses to overcome the automated link. From the perspective of the strength model, self-control, that is the capacity to inhibit the automated response, is a limited resource permitting individuals to inhibit their responses for a finite period until the resource becomes depleted limiting subsequent capacity for inhibition. An important feature of the self-control ‘resource’ is that it is ‘domain general’ i.e. it is a generalizable resource that enables individuals to control their behavior in multiple domains. This has been shown in many studies in which individuals engaged in a task that requires them to inhibit their self-control in one domain results in impaired response inhibition in another. Importantly in the current context, exerting self-control on laboratory-based tasks that require the inhibition of responses leads to reduced capacity to inhibit responses when presented with tempting behaviors in health-related domains such as resisting alcohol in social drinkers or tempting foods in people with low eating restraint. Consistent with this line of research, studies have revealed that training on tasks that require self-control i.e. the ability to inhibit responses will improve response inhibition capacity and provide individuals to inhibit cue-response pairings more effectively. And this effect also appears to be domain general, consistent with the strength model [78] and the reflective-impulsive model [97]. Training on self-control tasks which require regular inhibition of a pre-potent response is, therefore, hypothesised to improve generalized capacity for self-control as shown in previous studies [84,88]. With individuals who have sufficient motivation to reduce their alcohol intake, improving response inhibition capacity is expected to improve capacity to inhibit the temptation to drink. This may be relevant in situations where they may be tempted to drink more than usual. They can therefore bring their improved generalized capacity to bear on reducing their alcohol consumption is situations where they may be tempted to drink to excess. Their additional response inhibition capability afforded to them by virtue of the training would provide sufficient capacity to override the automatic, cue-driven response to stimuli to drink alcohol.

We therefore propose that self-control training will only have a substantive effect on behavioral outcomes if individuals are motivated to change their behavior. Participants whose self-control capacity has been trained, but have little or no motivation to reduce their alcohol consumption are less likely to direct the increased self-control capacity gleaned from training toward that particular behavior. They may direct their efforts toward behaviors to which they are more motivated instead. We therefore expect that the combined manipulation of self-control training and motivational imagery-based intervention components, namely, mental simulations, to be more effective in changing behavior than either of the components alone. Importantly, the factorial design adopted in the present study permits the evaluation of the independent and synergistic effects of each of the intervention components on alcohol consumption. In addition, we expect the intervention to be highly acceptable for use in public health promotion campaigns due to its low response burden and highly practical, accessible, and cost-effective means of delivering the intervention.

We will administer outcome measures of two forms of alcohol consumption in the current research: total alcohol consumption and frequency of ‘binge’ drinking. Our primary outcome variable in the current research is overall self-reported alcohol consumption by undergraduate students. We cannot expect students to curb their alcohol drinking altogether, so the target outcome identified for participants in the current study will be keeping alcohol consumption within the guideline limits specified by the Australia National Health and Medical Research Council (NHMRC). The NHMRC guideline limits on safe alcohol consumption are 14 standard drinks (each standard drink is equivalent to 12.5 ml of pure alcohol) per week. The guideline limits will be clearly outlined to participants in advance of the research commencing and our purpose was to ensure that students did not exceed this limit as it is associated with long-term (chronic) harm [4]. We do, however, recognise that the guidelines reflect limits aimed at reducing chronic harm and that other patterns of alcohol consumption prevalent in students may also present a serious threat to health. For example, the consumption of 14 standard drinks on a single occasion, once per week may mean that an individual’s consumption falls within NHMRC overall guideline limits, but would constitute increased risk of acute harm. High-risk single-session alcohol consumption, also known as ‘binge’ drinking, defined as consuming more than 4 standard drinks on a single drinking occasion by the NHMRC [4], is related to substantially increased health risks such as unintentional injury, increased probability of unplanned and unprotected sexual intercourse, and risk of being involved in violence and social disorder [9,99,100]. Given that students are more likely to engage in binge drinking than their non-student peers [10,101], binge drinking is considered an important risk factor for alcohol-related harm specific to this population. We will, therefore, include a secondary outcome variable, frequency of occasions of binge drinking, self-reported by participants to account for this risky pattern of alcohol consumption that is likely to be endemic in this population. The variable is defined as the number of single occasions in which an individual’s alcohol consumption exceeded 4 standard drinks [4].

The intervention will comprise two components, motivational and self-control training. The motivational component of the intervention will comprise a process and outcome mental simulation manipulation in which participants will be required to visualize the steps they need to take in order to reduce keep their alcohol consumption within guideline limits in the next four weeks and the outcomes they will achieve. This task has been adopted in previous studies and the standardized protocol adopted in these studies will be used [34]. Participants not allocated to receive the motivational intervention component will receive an irrelevant visualization task. The self-control training component will require participants to engage in an online Stroop color-naming task delivered online either by smartphone or personal computer for the duration of the subsequent four-week period. The Stroop task was developed and piloted previously and has been shown to effectively enhance self-control capacity over a four-week training period [85]. Participants allocated to the self-control training condition will receive a ‘challenging’ version of the Stroop task while participants in the control condition will receive an ‘easy’ version, which is not expected to lead to any substantive improvements on self-control capacity.

## Methods

### Study design

**Figure 1:**
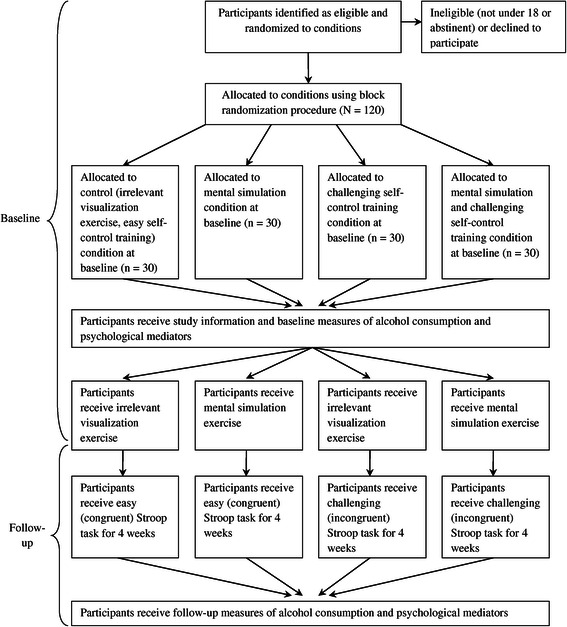
**Trial Flowchart.** The diagram illustrates the flow of participants through the proposed intervention.

The research will adopt a fully randomized-controlled 2 (mental simulation: mental simulation vs. control irrelevant visualization exercise) × 2 (self-control training: challenging Stroop task vs. easy Stroop task) between-participants factorial design. Mental simulation and self-control training manipulations will be the independent variables while self-reported alcohol consumption collected four-weeks after the initiation of the intervention will be the primary dependent variable. Baseline alcohol consumption and trait self-control will be included as covariates. Participants will be randomly allocated to one of the four intervention conditions according to a schedule generated by an online experimental randomising tool [102]. A participant flow diagram and overview of study design is provided in Figure 1. The study protocol has been submitted to, reviewed, and approved by Curtin University Human Research Ethics Committee.

### Participants

Participants will be undergraduate students from a large University in Western Australia. Students will be eligible to participate if that are 18 years or older and non-abstinent with respect to alcohol consumption to be eligible to participate in the study. Participants will be excluded if they are heavy or dependent drinkers as identified by the Fast Alcohol Screening Test (FAST) [103]. Participants will be recruited using email circular and notice board advertisements distributed throughout the University and from a dedicated participant pool for undergraduate psychology students incentivised by course credit or a prize draw for shopping vouchers. The study is described as an intervention to reduce alcohol consumption. Assuming a medium effect size for mental simulation interventions on behavior reported by Pham and Taylor [31] (*d* = 0.56) and an effect size of similar magnitude for the effects of self-control resource training reported in Hagger et al.’s [81] meta-analysis (*d* = .62), a power analysis using G*Power v3.1 [104] setting power at .80 and alpha at .05, estimates that we will require a total sample size of between 120 and 144 participants with between 30 and 36 participants in each intervention group. The variation in the sample size estimate is due to the use of the two different effect sizes to compute the power analysis, each of which gives a slightly different result.

### Procedure

On recruitment, participants will be required to attend an initial laboratory session with the experimenter at a mutually agreed time. Participants will be provided with an information sheet outlining their expectations and given the opportunity to ask questions about study requirements prior to completing and signing a consent form. They will then be asked to complete a brief demographic questionnaire and baseline self-report measures of alcohol consumption and psychological mediator variables. Next, participants will be given an envelope corresponding to the condition to which they have been allocated that contains instructions for the mental simulation or control manipulations. The envelopes will be prepared by an independent researcher using coded labels to represent the mental simulation manipulation and control irrelevant visualization task so that the experimenter will be blind to the intervention conditions until the end of the study. All participants will be presented with a written script with a generic introduction section providing a brief rationale for changing their alcohol behavior. The instructions will inform participants that the purpose of the study is to keep their alcohol consumption within nationally-recognized ‘safe’ guideline limits to promote better health and reduce adverse effects. A definition of NHMRC guideline limit for alcohol consumption of 14 standard drinks per week, a definition of a standard drink, and examples of volumes and measures of typical drinks that constitute a standard drink will be provided. This will be followed by instructions for the mental simulation or control irrelevant visualization exercise according to the randomly-allocated trial condition (see Additional file 1).

### Mental simulation intervention

Participants randomized to the mental simulation intervention, will be presented with written instructions in the form of a standardized script for the mental simulation exercise (Additional file 1). Participants will be instructed to imagine the steps they will take to reduce their alcohol consumption and the salient outcomes they expect to achieve. The instructions will be adapted from Pham and Taylor’s [31] ‘process’ and ‘outcome’ mental simulation scripts. Participants will be required to follow the instructions on the sheet and perform the metal simulation exercise with their eyes closed. Then they will be asked to write about their experience with the visualization exercise in the space provided on the sheet and to memorize it. Written responses will be content analysed to check for compliance with the task. The exercise is expected to take no longer than 10 minutes. Participants randomized to the control condition will receive written instructions in the form of a standardized script for an irrelevant visualization exercise identical in procedure to the mental simulation exercise (Additional file 1). Instead of visualizing reducing their alcohol consumption, however, participants will be required to imagine a recent visit to the cinema or shopping centre. They will also be asked to write down their experiences on the sheet provided. The purpose of the control task is to maintain an equivalent information load to the participants allocated to the mental simulation condition.

### Self-control training

We will use regular practice on an online version of the Stroop color-naming task which will be delivered on the participant’s smartphone as a means to train and improve self-control resource capacity. As a fall back, in case the participant does not have access to a smartphone or does not own a smartphone, we will give participants the opportunity to conduct their self-control training on their personal computer using their web browser. Participants will be randomly allocated to an intervention group that engages in practice on using a ‘challenging’ version of the Stroop task for the duration of the intervention while participants allocated to the comparison group will engage in regular practice on an ‘easy’ version of the task that is not proposed to have any substantive effect on training self-control. Participants will practice on the task twice per day for the four weeks duration of the intervention. The tasks are similar to those developed for use in a previous study [85]. The challenging version of the task requires participants to respond to a series of word items in which the word meaning and the color of the ink in which it is written are incongruent (e.g., the word “green” written in red ink). The easy version is modified such that the word meaning and ink colour are congruent. There are equal numbers of word items with four possible colors (“red”, “green”, “yellow”, and “blue”) presented in random order. Participants will be given instructions on how to perform the task on a smartphone or personal computer during the initial visit to the laboratory. Participants will be offered the opportunity to use a generic smartphone loaned to them by the experimental team, their own smartphone, or their personal computer to engage in the training tasks. Participants will be given ample opportunity to familiarize themselves with the task and to ask any questions. Participants will be informed that they will be prompted to engage in the task via a text message sent to their smartphone or an email to their regular email address containing a URL link. Upon clicking the URL link, participants will be directed to a website to complete the version of Stroop color-naming task corresponding to the condition to which they will be allocated. Participants will be presented with a series of 238 color-word items presented in seven 34-item blocks on their screen with each item presented for 2000 milliseconds (ms). Participants will use a response panel presented on the smartphone touchscreen or their computer keyboard to identify the colour in which the word is written rather than respond to the word meaning. Participants will have 800 ms to provide a response by choosing the colour-word response options. Response latencies for each item will be logged along with errors. If no response is given, the response will be logged as “unanswered” and the next colour-word item will be presented.

The Stroop task is expected to approximately 5 minutes. Participants will complete the task to which they have been assigned immediately after the mental simulation task in the initial session and then repeat the Stroop task twice per day when prompted, at 7 am and 5 pm, throughout the four-week intervention period. All participants’ Stroop data will be uploaded to, and stored on, a remote computer server for subsequent retrieval to compliance analysis.

Participants will be reminded to practice the Stroop task once per week via emails and/or text messages throughout the four-week intervention period. After four weeks participants will be sent a series of reminder emails to prompt them to complete follow-up dependent measures of self-reported alcohol consumption and follow-up measures of psychological mediator variables, identical to those administered at baseline. Thereafter, participants will receive a full debrief and provided with an opportunity to receive the final group-level results of the intervention.

### Primary and secondary outcome measures

Our primary outcome measure is participants’ self-reported alcohol consumption over the previous four weeks consistent with NHMRC guidelines [4]. Our secondary dependent variable is participants’ self-reported number of binge drinking occasions over the previous four weeks. Both primary and secondary outcome measures will administered at baseline and at follow-up. Participants will be asked to recall and report the absolute number of standard drinks they had consumed and the number of occasions when they engaged in binge drinking, that is, exceeding more than 4 standard drinks on a single drinking occasion, per week over the previous four weeks [34,105]. The self-report measures are based on the time-line follow back technique which has been shown to provide precise estimates of alcohol drinking [106]. This measure uses a number of techniques to aid recall such as linking alcohol drinking with significant events. In addition, the FAST will be administered to students recruited to the study at baseline [103]. The measure comprises four items (e.g. “How often during the last year have you failed to do what was normally expected of you because of drinking?”) and has demonstrated adequate validity and reliability to assess the extent of heavy and dependent drinking. FAST scores will be used as a means to screen participants for heavy alcohol consumption that is indicative of alcohol dependency. Participants identified as heavy or dependent drinkers will be excluded from the study prior to engaging in the protocol. Participants identified as heavy or dependent drinkers by the FAST will be provided with a leaflet providing advice on how to seek professional help for their alcohol consumption.

### Measures of psychological mediator variables

In keeping with research examining the effects of the intervention components on behavior, we will include measures of key psychological variables that are likely to mediate the impact of the motivational and self-control intervention techniques on alcohol consumption. Mental simulations are likely to be mediated by social cognitive variables related to motivation. In keeping with previous research that has demonstrated significant effects of mental simulations on variables form the theory of planned behavior, we will measure participants’ intentions, attitudes, perceived behavioral control, and subjective norms toward alcohol consumption. Standardized measures of the theory of planned behavior constructs used in previous studies will be adopted [42,107]. Intentions will be assessed with three items (e.g. “I intend to keep my alcohol drinking to within safe limits on each individual occasion or session over the next four weeks”) rated on six-point Likert-type scales ranging from 1 (*extremely unlikely*) to 6 (*extremely likely*). Attitudes will be assessed using five semantic-differential items (e.g. *enjoyable-unenjoyable*, *important-unimportant*) on six-point scales in response to a common stem (“For me, keeping my alcohol drinking to within safe limits on each individual occasion or session over the next four weeks is…”). Perceived behavioral control will be measured using three items (e.g. “How confident are you that you can keep your alcohol drinking to within safe limits on each individual occasion or session over the next four weeks?”) on six-point Likert-type scales ranging from 1 (*no control at all*) to 6 (*complete control*). Subjective norms will be assessed using three items (e.g. “Most people I know would approve of me keeping my alcohol drinking to within safe limits on each individual occasion or session over the next four weeks.”) on six-point Likert-type scales ranging from 1 (*disagree*) to 6 (*agree*). We will also assess generalized motivation to reduce alcohol consumption based on measures identified in previous research [34,107,108]. The motivation measure will adopt three items (e.g. “How motivated are you to keep your alcohol drinking to within safe limits on each individual occasion or session over the next four weeks?”) with responses made on six-point Likert-type scales ranging from 1 (*not at all motivated*) to 6 (*extremely motivated*).

We also expect that the effect of self-control training on alcohol consumption will be mediated by perceptions of subjective self-control capacity. We will therefore include a modified self-report measure of self-control to reflect current self-control capacity. Specifically, we will adopt the state self-control capacity scale to measure participants current self-control reserve (Ciarocco, Twenge, Muraven, & Tice, 2011). The scale comprises 25-items (e.g., *“I feel discouraged”*) assessed on seven-point Likert-type scales ranging from 1 (*not true*) to 7 (*very true*). In addition, we will assess dispositional levels of self-control using Tangney et al.’s [73] self-control questionnaire. The short version of the scale will be used which comprises 13-items (e.g., *“I am lazy”*) assessed on five-point Likert scales ranging from 1 (*not true at all*) to 5 (*very true*). The trait scale will only be administered to participants in the introductory session and not at follow-up.

### Data analysis

The first part of the intervention will involve testing intervention compliance. We will evaluate participants’ compliance with the mental simulation manipulation by conducting a content analysis of participants’ scripts written during the course of the intervention. We will evaluate compliance with the self-control training by examining participants’ level of engagement with the Stroop tasks administered over the course of the four-week intervention period. The use of online delivery of the self-control training intervention will enable us to analyze the percentage compliance with the task (including feigned compliance, engaging in the task but providing meaningless responses) and any improvements in Stroop performance over the course of the intervention relative to baseline. This will also enable us to evaluate whether there were differences in compliance across the challenging and easy self-control tasks. Similar to previous studies [14,34,108,109], we will conduct a 2 (self-control training: challenging vs. easy) × 4 (test week: 1, 2, 3 or 4) mixed-model ANOVA with repeated measures on the second factor to test for changes in Stroop performance over the course of the intervention. We will use averaged weekly Stroop response latencies as the dependent variable and hypothesize statistically-significant differences of medium effect sizes across intervention groups with longer latencies for participants receiving the challenging Stroop task, but we expect a statistically-significant training condition by time interaction effect of medium size such that the differences diminish over the course of the intervention as participants receiving the challenging task improve their self-control while participants receiving the easy task exhibit little or no improvement.

Data on the primary dependent variable of alcohol consumption will be analysed using a 2 (mental simulation: mental simulation vs. no mental simulation) × 2 (self-control training: challenging vs. easy) factorial between-participants analysis of covariance (ANCOVA) with alcohol consumption at follow-up as the dependent variable and baseline alcohol consumption and trait self-control as covariates. The analysis permits the main and interactive effects of mental simulation and self-control training intervention components. We predict statistically-significant main effects of medium size of mental simulation and self-control training on alcohol consumption, but also expect a statistically-significant, medium-sized interaction effect such that participants receiving the mental simulation and challenging self-control training components will report significantly lower levels of alcohol consumption than participants receiving either of the intervention components alone and participants that received neither manipulation.

We will also conduct mediation analyses to evaluate the effectiveness of the intervention components on alcohol consumption. This will be conducted using moderated linear regression analyses with indirect effects reproduced using Preacher, Curran, and Bauer’s [110] asymptotic bootstrapped algorithms and the bias-corrected bootstrap confidence interval to assess the statistical significance of the effects [111]. Specifically, we will develop binary dummy-coded variables to represent the effects of the mental simulation (1 = received mental simulation, 0 = received irrelevant visualization strategy) and self-control training (1 = received challenging Stroop task, 0 = received easy Stroop task) interventions with an additional variable computed to represent the interaction of the two. The primary outcome variable, follow-up alcohol consumption, will be regressed on these coded variables in the first instance to ascertain direct effects. This will be followed by analyses including multiple mediators of the effects including residualized change scores for the baseline and follow-up measures of the theory of planned behavior variables (intentions, subjective norms, attitudes, perceived behavioral control) and motivation as mediators of the mental simulation intervention and residualized change scores in state perceived self-control between baseline and follow-up as a mediator of the effect of the self-control training intervention. The unstandardized effects and confidence intervals will be used as input for Preacher et al.’s [110] bootstrapped algorithm computational tool to compute the indirect effects. We predict statistically-significant, medium-sized multiple mediation of the effects of the mental simulation intervention on follow-up alcohol consumption by the theory of planned behavior and motivational change scores and significant mediation of the effect of self-control training on alcohol consumption by state self-control change scores. Trait self-control will be included in the analyses as a control variable.

## Discussion

Excessive consumption of alcohol among young people has been recognized as a significant public health issue due to its association with numerous health, social, and economic problems [1]. Undergraduate students tend to be a particularly high-risk group as they frequently engage in risky patterns of alcohol consumption such as binge-drinking [5-7]. Behavioral interventions have been shown to be effective in reducing alcohol consumption in young people e.g., [14,108], but few have reported the theoretical basis of the intervention and its components, tested the independent and interactive effects of the techniques aimed at reducing alcohol consumption, and identified they key mediators of the intervention effects.

The present study aims to conduct a four-week theory-based intervention using leading behavior-change techniques to reduce alcohol consumption at follow-up among undergraduate students. The intervention will adopt motivational and self-control training behavior-change techniques derived from psychological theory and use a randomized-controlled factorial design to examine the unique and interactive effect of each technique in on alcohol consumption. According to theory, we hypothesize that participants receiving both intervention components should exhibit the lowest levels of alcohol consumption at follow-up relative to participants receiving each of the intervention conditions alone and participants receiving neither of the components. In addition, the research will also evaluate the theoretically-relevant mediating variables that explain the effect of the motivational and self-control training intervention components on alcohol consumption. Specifically, we expect the motivational intervention component to be mediated by social cognitive variables including intentions, attitudes, and motivation and the self-control intervention to be mediated by perceived self-control resource availability.

The research will make a unique contribution to knowledge regarding interventions to reduce alcohol consumption and binge drinking occasions in undergraduate students because it will (a) address the limitations of previous research using multiple intervention techniques concurrently by identifying the unique and interactive effects of two isolated and independent intervention techniques (mental simulation and self-control training) to reduce alcohol consumption through the use of a factorial design [20,112]; (b) assist in identifying the mechanisms behind motivational and self-control training components and their interaction on alcohol consumption by testing the effects of key proposed mediating variables [18]; (c) adopt methods that allow for the efficacy of the intervention to be evaluated including written responses to the mental simulation motivational intervention [108] and using an objective means to manage compliance with the programme of self-control resource training via an online training task [85]; and (d) extend knowledge of the causal effects of an intervention aimed at affecting *change* in health behavior through motivational and self-control related variables beyond that which can be inferred from correlational [113] and panel [114] designs.

### Implications

The proposed study has important implications for public health specialists and researchers designing behavioral interventions. The research is relevant to specialists in public health because it will demonstrate the effectiveness of brief interventions including motivational and self-control training components in reducing undergraduate students’ alcohol consumption, the primary outcome variable. This is likely to contribute to initiatives to reduce maladaptive outcomes associated with excessive alcohol consumption in this population. Importantly, the intervention techniques have low response burden and cost and are administered using efficient and effective delivery methods that are readily available including the internet and smartphones. The research is of interest to researchers as it will provide detail on the individual and interactive components of motivational and self-control training on alcohol consumption and also demonstrate the key mediators of the effects. This is important as it will assist in elucidating the ‘active ingredients’ of the intervention and the mechanisms by which they exert their effects.

## Additional file

Additional file 1:
**Intervention materials including introduction, mental simulation, and irrelevant visualization scripts.**

## Abbreviations

NHMRC: National health and medical research council
FAST: Fast alcohol screening test

## References

[1] AIHW (2011). 2010 National Drug Strategy Household Survey report.

[2] Pennay A, Lubman DI, Maclean S (2011). Risky drinking among young Australians - Causes, effects and implications for GPs. Aust Fam Physician.

[3] Manning M, Smith C, Mazerolle P (2013). The societal costs of alcohol misuse in Australia (No. 454).

[4] NHMRC (2009). Australian Guidelines to reduce health risks from drinking alcohol.

[5] Hallett J, Howat PM, Maycock BR, McManus A, Kypri K, Dhaliwal SS (2012). Undergraduate student drinking and related harms at an Australian university: Web-based survey of a large random sample. BMC Public Health.

[6] Naimi TS, Brewer RD, Mokdad A, Denny C, Serdula MK, Marks JS (2003). Binge drinking among US adults. JAMA.

[7] Australian Bureau of Statistics (2008). Risk taking by young people.

[8] Karam E, Kypri K, Salamoun M (2007). Alcohol use among college students: An international perspective. Curr Opin Psychiatry.

[9] Mundt MP, Zakletskaia LI, Fleming MF (2009). Extreme college drinking and alcohol-related injury risk. Alcohol Clin Exp Res.

[10] Gill JS (2002). Reported levels of alcohol consumption and binge drinking within the UK undergraduate student population over the last 25 years. Alcohol Alcohol.

[11] Thombs DL, Olds RS, Bondy SJ, Winchell J, Baliunas D, Rehm J (2009). Undergraduate drinking and academic performance: A prospective investigation with objective measures. J Stud Alcohol Drugs.

[12] Martinez JA, Sher KJ, Wood PK (2008). Is heavy drinking really associated with attrition from college? The alcohol-attrition paradox. Psychol Addict Behav.

[13] Fager JH, Melnyk MB (2004). The effectiveness of intervention studies to decrease alcohol use in college undergraduate students: An integrative analysis. Worldviews Evid Based Nurs.

[14] Hagger MS, Lonsdale AJ, Chatzisarantis NLD (2012). A theory-based intervention to reduce alcohol drinking in excess of guideline limits among undergraduate students. Br J Health Psychol.

[15] Murgraff V, Abraham C, McDermott M (2007). Reducing Friday alcohol consumption among moderate, women drinkers: Evaluation of a brief evidence-based intervention. Alcohol Alcohol.

[16] French DP, Cooke R (2012). Using the theory of planned behaviour to understand binge drinking: The importance of beliefs for developing interventions. Br J Health Psychol.

[17] Jessop DC, Wade J (2008). Fear appeals and binge drinking: A terror management theory perspective. Br J Health Psychol.

[18] Hagger MS, Chatzisarantis NLD (2014). An integrated behavior-change model for physical activity. Exerc Sport Sci Rev.

[19] Peters G-JY, de Bruin M, Crutzen R. Everything should be as simple as possible, but no simpler: Towards a protocol for accumulating evidence regarding the active content of health behaviour change interventions. Health Psychol Rev. 2015: doi:10.1080/17437199.17432013.17848409. 10.1080/17437199.2013.848409 PMC4376231 25793484

[20] Michie S (2008). What works and how? Designing more effective interventions needs answers to both questions. Addiction.

[21] Michie S, Ashford S, Sniehotta FF, Dombrowski SU, Bishop A, French DP (2011). A refined taxonomy of behaviour change techniques to help people change their physical activity and healthy eating behaviours: The CALO-RE taxonomy. Psychol Health.

[22] Abraham C, Michie S (2008). A taxonomy of behavior change techniques used in interventions. Health Psychol.

[23] Michie S, Whittington C, Hamoudi Z, Zarnani F, Tober G, West R (2012). Identification of behaviour change techniques to reduce excessive alcohol consumption. Addiction.

[24] Abraham C. Mapping modifiable mechanisms in health promotion research: a commentary on Sniehotta, Presseau, and Araújo-Soares. Health Psychol Rev. 2015: doi:10.1080/17437199.17432014.17905967. 10.1080/17437199.2014.905967 26209204

[25] Crisp RJ, Birtel MD, Meleady R (2011). Mental simulations of social thought and action. Pers Soc Psychol Rev.

[26] Vasquez NA, Buehler R (2007). Seeing future success: Does imagery perspective influence achievement motivation?. Pers Soc Psychol Bull.

[27] Bandura A (1977). Self-efficacy: Toward a unifying theory of behavioral change. Psychol Rev.

[28] Bandura A (1986). Social foundations of thought and action: A social-cognitive theory.

[29] Knauper B, McCollam A, Rosen-Brown A, Lacaille J, Kelso E, Roseman M (2011). Fruitful plans: Adding targeted mental imagery to implementation intentions increases fruit consumption. Psychol Health.

[30] Knauper B, Roseman M, Johnson PJ, Krantz LH (2009). Using mental imagery to enhance the effectiveness of implementation intentions. Curr Psychol.

[31] Pham LB, Taylor SE (1999). From thought to action: Effects of process- versus outcome-based mental simulations on performance. Pers Soc Psychol Bull.

[32] Escalas JE, Luce MF (2003). Process versus outcome thought focus and advertising. J Consum Psychol.

[33] Armitage CJ, Reidy JG (2012). Evidence that process simulations reduce anxiety in patients receiving dental treatment: Randomized exploratory trial. Anxiety Stress Coping.

[34] Hagger MS, Lonsdale AJ, Chatzisarantis NLD (2011). Effectiveness of a brief intervention using mental simulations in reducing alcohol consumption in corporate employees. Psychol Health Med.

[35] Webb TL, Sheeran P (2006). Does changing behavioral intentions engender behavior change? A meta-analysis of the experimental evidence. Psychol Bull.

[36] Williams DM, Rhodes RE. The confounded self-efficacy construct: conceptual analysis and recommendations for future research. Health Psychol Rev. 2015: doi:10.1080/17437199.17432014.17941998. 10.1080/17437199.2014.941998 PMC4326627 25117692

[37] Hagger MS, Keatley DA, Chan DK-C, Eklund RC, Tenenbaum GT (2014). CALO-RE Taxonomy of Behavior Change Techniques. Encyclopedia of Sport and Exercise Psychology.

[38] Michie S, West R (2013). Behaviour change theory and evidence: A presentation to Government. Health Psychol Rev.

[39] Hagger MS, Chatzisarantis NLD, Barkoukis V, Wang CKJ, Hein V, Pihu M (2007). Cross-cultural generalizability of the Theory of Planned Behavior among young people in a physical activity context. J Sport Exerc Psychol.

[40] Ajzen I. The theory of planned behavior is alive and well, and not ready to retire. Health Psychol Rev. 2015. doi:10.1080/17437199.2014.883474. 10.1080/17437199.2014.883474 26209198

[41] Hagger MS, Anderson M, Kyriakaki M, Darkings S (2007). Aspects of identity and their influence on intentional behaviour: Comparing effects for three health behaviours. Pers Indiv Diff.

[42] Hagger MS, Chatzisarantis NLD (2006). Self-identity and the theory of planned behaviour: Between-and within-participants analyses. Br J Soc Psychol.

[43] Sniehotta FF, Presseau J, Araújo-Soares V (2014). Time to retire the Theory of Planned Behaviour. Health Psychol Rev.

[44] Noar SM, Head KJ (2014). Mind the gap: bringing our theories in line with the empirical data – a response to commentaries. Health Psychol Rev.

[45] Mankarious E, Kothe E. A meta-analysis of the effects of measuring theory of planned behaviour constructs on behaviour within prospective studies. Health Psychol Rev. 2015:doi:10.1080/17437199.17432014.17927722. 10.1080/17437199.2014.927722 26209208

[46] Cooke R, Dahdah M, Norman P, French DP. How well does the theory of planned behaviour predict alcohol consumption? A systematic review and meta-analysis. Health Psychol Rev. 2015: doi:10.1080/17437199.17432014.17947547. 10.1080/17437199.2014.947547 PMC4867851 25089611

[47] Barkoukis V, Hagger MS, Lambropoulos G, Torbatzoudis H (2010). Extending the trans-contextual model in physical education and leisure-time contexts: Examining the role of basic psychological need satisfaction. Br J Educ Psychol.

[48] Hein V, Hagger MS (2007). Global self-esteem, goal achievement orientations and self-determined behavioural regulations in physical education setting. J Sports Sci.

[49] Hagger MS, Sultan S, Hardcastle SJ, Chatzisarantis NLD (2015). Perceived autonomy support and autonomous motivation toward mathematics activities in educational and out-of-school contexts is related to mathematics homework behavior and attainment. Contemp Educ Psychol.

[50] Chatzisarantis NLD, Hagger MS, Smith B (2007). Influences of perceived autonomy support on physical activity within the theory of planned behavior. Eur J Soc Psychol.

[51] Chatzisarantis NLD, Hagger MS, Smith B, Sage LD (2006). The influences of intrinsic motivation on execution of social behaviour within the theory of planned behaviour. Eur J Soc Psychol.

[52] Hagger MS, Chatzisarantis NLD (2012). Transferring motivation from educational to extramural contexts: A review of the trans-contextual model. Eur J Psychol Educ.

[53] Jacobs N, Hagger MS, Streukens S, De Bourdeaudhuij I, Claes N (2011). Testing an integrated model of the Theory of Planned Behaviour and Self-Determination Theory for different energy-balance related behaviours and intervention intensities. Br J Health Psychol.

[54] Chatzisarantis NLD, Hagger MS, Brickell T (2008). Using the construct of perceived autonomy support to understand social influence in the theory of planned behavior. Psychol Sport Exerc.

[55] Chan DKC, Spray C, Hagger MS (2011). Treatment motivation for rehabilitation after a sport injury: Application of the trans-contextual model. Psychol Sport Exerc.

[56] Hamilton K, Cox S, White KM (2012). Testing a model of physical activity among mothers and fathers of young children: integrating self-determined motivation, planning, and theory of planned behavior. J Sport Exerc Psychol.

[57] Andersson EK, Moss TP (2011). Imagery and implementation intention: A randomised controlled trial of interventions to increase exercise behaviour in the general population. Psychol Sport Exerc.

[58] Armitage CJ, Reidy JG (2008). Use of mental simulations to change theory of planned behaviour variables. Br J Health Psychol.

[59] Hagger MS, Luszczynska A (2014). Implementation intention and action planning interventions in health contexts: State of the research and proposals for the way forward. Appl Psychol-Health Well Being.

[60] Duncan LR, Rodgers WM, Hall CR, Wilson PM (2011). Using imagery to enhance three types of exercise self-efficacy among sedentary women. Appl Psychol-Health Well Being.

[61] Kim BH, Newton RA, Sachs ML, Glutting JJ, Glanz K (2012). Effect of guided relaxation and imagery on falls self-efficacy: A randomized controlled trial. J Am Geriatr Soc.

[62] de Ridder DTD, Lensvelt-Mulders G, Finkenauer C, Stok FM, Baumeister RF (2012). Taking stock of self-control: A meta-analysis of how trait self-control relates to a wide range of behaviors. Pers Soc Psychol Rev.

[63] Hagger MS (2010). Self-regulation: An important construct in health psychology research and practice. Health Psychol Rev.

[64] Hagger MS, Wood C, Stiff C, Chatzisarantis NLD (2009). The strength model of self-regulation failure and health-related behavior. Health Psychol Rev.

[65] Muraven M, Baumeister RF (2000). Self-regulation and depletion of limited resources: Does self-control resemble a muscle?. Psychol Bull.

[66] Hagger MS, Leung CM, Leaver E, Esser K, te Pas N, Keatley DA (2013). Cue-induced smoking urges deplete cigarette smokers’ self-control resources. Ann Beh Med.

[67] Hagger MS, Chatzisarantis NLD (2013). The sweet taste of success: The presence of glucose in the oral cavity moderates the depletion of self-control resources. Pers Soc Psychol Bull.

[68] Hagger MS, Panetta G, Leung C-M, Wong GG, Wang JCK, Chan DK-C (2013). Chronic inhibition, self-control and eating behavior: Test of a ‘resource depletion’ model. PLoS One.

[69] Hagger MS, Rebar AL, Mullan BA, Lipp OV, Chatzisarantis NLD. The subjective experience of habit captured by self-report indexes may lead to inaccuracies in the measurement of habitual action. Health Psychol Rev 2015. doi: 10.1080/17437199.17432014.17959728. 10.1080/17437199.2014.959728 25189762

[70] Hagger MS, Chatzisarantis NLD (2014). It is premature to regard the ego-depletion effect as ‘too incredible’. Front Psychol.

[71] Loftus AM, Yalcin O, Baughman FD, Vanman EJ, Hagger MS. The impact of transcranial direct current stimulation on inhibitory control in young adults. Brain Behav. 2015: doi:10.1002/brb1003.1332. 10.1002/brb3.332 PMC4389055 25874165

[72] Rebar AL, Loftus AM, Hagger MS (2015). Cognitive control and the non-conscious regulation of health behavior. Front Hum Neurosci.

[73] Tangney JP, Baumeister RF, Boone AL (2004). High self-control predicts good adjustment, less pathology, better grades, and interpersonal success. J Pers.

[74] Hagger MS (2013). The multiple pathways by which self-control predicts behavior. Front Psychol.

[75] Hagger MS (2014). Where does sleep fit in models of self-control and health behaviour?. Stress Health.

[76] Hagger MS (2014). The multiple pathways by which trait self-control predicts health behavior. Ann Behav Med.

[77] Hagger MS. Implicating self-control in the mechanism by which implementation intentions reduce stress-induced unhealthy eating: A comment on O’Connor et al. ann behav med 2015. 10.1007/s12160-014-9678-8 25582988

[78] Baumeister RF, Bratslavsky E, Muraven M, Tice DM (1998). Ego depletion: Is the active self a limited resource?. J Pers Soc Psychol.

[79] Muraven M, Tice DM, Baumeister RF (1998). Self-control as a limited resource: Regulatory depletion patterns. J Pers Soc Psychol.

[80] Hagger MS, Wood C, Stiff C, Chatzisarantis NLD (2010). Self-regulation and self-control in exercise: The strength-energy model. Int Rev Sport Exerc Psychol.

[81] Hagger MS, Wood C, Stiff C, Chatzisarantis NLD (2010). Ego depletion and the strength model of self-control: A meta-analysis. Psychol Bull.

[82] Chatzisarantis NLD, Hagger MS (2015). Unsuccessful attempts to replicate effects of self control operations and glucose on ego-depletion pose an interesting research question that demands explanation. Appetite.

[83] Chatzisarantis NLD, Hagger MS (2015). Illusionary delusions. Willingness to exercise self-control can mask effects of glucose on self-control performance in experimental paradigms that use identical self-control tasks. Appetite.

[84] Muraven M, Baumeister RF, Tice DM (1999). Longitudinal improvement of self-regulation through practice: Building self-control strength through repeated exercise. J Soc Psychol.

[85] Cranwell J, Benford S, Houghton R, Golembewksi M, Fischer JF, Hagger MS (2013). Increasing self-regulatory energy using an Internet-based training application delivered by smartphone technology. Cyberpsychol Behav Soc Netw.

[86] Hui S-KA, Wright RA, Stewart CC, Simmons A, Eaton B, Nolte RN (2009). Performance, cardiovascular, and health behavior effects of an inhibitory strength training intervention. Motiv Emot.

[87] Oaten M, Cheng K (2006). Improved self-control: The benefits of a regular program of academic study. Basic Appl Soc Psychol.

[88] Oaten M, Cheng K (2006). Longitudinal gains in self-regulation from regular physical exercise. Br J Health Psychol.

[89] Houben K (2011). Overcoming the urge to splurge: Influencing eating behavior by manipulating inhibitory control. J Behav Ther Exp Psychiatry.

[90] Houben K, Jansen A (2011). Training inhibitory control. A recipe for resisting sweet temptations. Appetite.

[91] Houben K, Wiers RW, Jansen A (2011). Getting a Grip on Drinking Behavior: Training Working Memory to Reduce Alcohol Abuse. Psychol Sci.

[92] Houben K, Nederkoorn C, Wiers RW, Jansen A (2011). Resisting temptation: Decreasing alcohol-related affect and drinking behavior by training response inhibition. Drug Alcohol Depend.

[93] Mullan BA, Wong C, Allom V, Pack SL (2011). The role of executive function in bridging the intention-behaviour gap for binge-drinking in university students. Addict Behav.

[94] Allom V, Mullan BA, Hagger MS (2015). Does inhibitory control training improve behavior regulation? A meta-analysis. Unpublished manuscript.

[95] Muraven M, Slessareva E (2003). Mechanisms of self-control failure: Motivation and limited resources. Pers Soc Psychol Bull.

[96] Moller AC, Deci EL, Ryan RM (2006). Choice and ego depletion: The moderating role of autonomy. Pers Soc Psychol Bull.

[97] Strack F, Deutsch R (2004). Reflective and impulsive determinants of social behavior. Pers Soc Psychol Rev.

[98] Metcalfe J, Mischel W (1999). A hot/cool-system analysis of delay of gratification: The dynamics of willpower. Psychol Rev.

[99] Plant MA, Plant ML, Miller P, Gmel G, Kuntsche S (2009). The social consequences of binge drinking: A comparison of young adults in six European countries. J Addict Dis.

[100] Nelson TF, Xuan ZM, Lee H, Weitzman ER, Wechsler H (2009). Persistence of heavy drinking and ensuing consequences at heavy drinking colleges. J Stud Alcohol Drugs.

[101] Bailer J, Stubinger C, Dressing H, Gass P, Rist F, Kuhner C (2009). Increased prevalence of problematic alcohol consumption in university students. Psychother Psychosom Med Psychol.

[102] Urbaniak GC, Plous S, Lestik M: Research randomiser. 2007:Accessed 20 January 2010 from www.randomizer.org.

[103] Hodgson RJ, Alwyn T, John B, Thom B, Smith A (2002). The fast alcohol screening test. Alcohol Alcohol.

[104] Faul F, Erdfelder E, Lang A-G, Buchner A (2007). G*Power 3: A flexible statistical power analysis program for the social, behavioral, and biomedical sciences. Behav Res Methods.

[105] Hagger MS, Lonsdale A, Hein V, Koka A, Lintunen T, Pasi HJ (2012). Predicting alcohol consumption and binge drinking in company employees: An application of planned behaviour and self-determination theories. Br J Health Psychol.

[106] Sobel ME, Leinhardt S (1982). Asymptotic confidence intervals for indirect effects in structural equation models. Sociological Methodology.

[107] Hagger MS, Chatzisarantis NLD, Harris J (2006). The process by which relative autonomous motivation affects intentional behavior: Comparing effects across dieting and exercise behaviors. Motiv Emot.

[108] Hagger MS, Lonsdale A, Koka A, Hein V, Pasi H, Lintunen T (2012). An intervention to reduce alcohol consumption in undergraduate students using implementation intentions and mental simulations: A cross-national study. Int J Behav Med.

[109] Hardcastle SJ, Taylor AH, Bailey MP, Harley RP, Hagger MS (2013). Effectiveness of a motivational interviewing intervention on weight loss, physical activity and cardiovascular disease risk factors: A randomised controlled trial with a 12-month post-intervention follow-up. Int J Behav Nutr Phys Act.

[110] Preacher KJ, Curran PJ, Bauer DJ (2006). Computational tools for probing interaction effects in multiple linear regression, multilevel modeling, and latent curve analysis. J Educ Behav Stat.

[111] Hayes AF, Scharkow M (2013). The relative trustworthiness of inferential tests of the indirect effect in statistical mediation analysis: Does method really matter?. Psychol Sci.

[112] Michie S, Johnston M (2012). Theories and techniques of behaviour change: Developing a cumulative science of behaviour change. Health Psychol Rev.

[113] Hagger MS, Chatzisarantis NLD (2009). Assumptions in research in sport and exercise psychology. Psychol Sport Exerc.

[114] Lindwall M, Larsmann P, Hagger MS (2011). The reciprocal relationship between physical activity and depression in older European adults: A prospective cross-lagged panel design using SHARE data. Health Psychol.

