# Supplementary material for: A theory-based behavior-change intervention to reduce alcohol consumption in undergraduate students: Trial protocol
Source: BMC Public Health. 2015 Mar 31;15:306. doi: 10.1186/s12889-015-1648-y (PMC4392782; doi:10.1186/s12889-015-1648-y)
Supplement: Additional file 1: — Intervention materials including introduction, mental simulation, and irrelevant visualization scripts. [file 12889_2015_1648_MOESM1_ESM.docx]

**Additional file 1**

**Introductory Passage (All Participants)**

“The National Health and Medical Research Council recommends that safe limits for drinking alcohol is 14 standard drinks per week and no more than 4 standard drinks in any single session. Drinking above these safe limits could lead to some health conditions in the long run. Considering these health messages, we would like you to try to keep your regular alcohol intake so that it is within recommended limits on each individual occasion or session over the next four weeks.”

**Mental Simulation Manipulation (Mental Simulation Condition Only)**

“You are now asked to visualize yourself drinking alone or drinking with your friends or family members in a way that you are trying to keep your alcohol intake within safe limits, and imagine how you would feel. Imagine what you would do to keep your drinking within safe limits. Imagine how much effort and willpower it has taken to maintain your drinking within safe limits. It is very important that you see yourself actually trying to keep your alcohol intake within safe limits on each individual occasion or session over the next four weeks, and keep that picture on your mind. Now, rehearse the above instructions with your eyes closed. Once you have done so, please type in the space below how you imagine you will feel if you achieve your goal of keeping your alcohol intake within safe limits on each individual occasion or session over the next month.”

“You are now asked to visualize yourself having achieved your goal of keeping your alcohol intake to within safe limits on each individual occasion or session over the next three months, and imagine how you would feel. Imagine how much effort and willpower it has taken to achieving your goal of keeping your alcohol intake to within safe limits on each occasion or session and that you have successfully managed to do it. Imagine how satisfied you will feel. It is very important that you see yourself actually keeping your alcohol intake to within safe limits on each occasion or session over the next months and keep that picture on your mind. One you have done so, please type in the space below how you imagine you will feel if you achieve your goal of keeping your alcohol intake within safe limits on each individual occasion or session over the next month.”

**Irrelevant Visualization Exercise (Control Condition Only)**

“You are now asked to visualize your most recent trip to the cinema or to the shopping mall alone or with your friends or family members, and imagine how you would feel. Imagine which movie you have watched or what you have done at the shopping mall. It is very important that you see yourself actually watching movie in the cinema or shopping in the shopping mall, and keep that picture in your mind. Now, rehearse the above instructions with your eyes closed. Once you have done so, please type in the space below how you would feel while watching movie or what you have done at the shopping mall.”
